# The epidemiology, molecular pathogenesis, diagnosis, and treatment of maturity-onset diabetes of the young (MODY)

**DOI:** 10.1186/s40842-020-00112-5

**Published:** 2020-11-04

**Authors:** Ken Munene Nkonge, Dennis Karani Nkonge, Teresa Njeri Nkonge

**Affiliations:** 1 University of Nairobi, P.O. Box 30197, Nairobi, Kenya; 2 McMaster University, Hamilton, Ontario L8S 4L8 Canada

**Keywords:** Beta cell, Diagnosis, MODY, Monogenic diabetes, Pathogenesis, Prevalence, Treatment

## Abstract

**Background:**

The most common type of monogenic diabetes is maturity-onset diabetes of the young (MODY), a clinically and genetically heterogeneous group of endocrine disorders that affect 1–5% of all patients with diabetes mellitus. MODY is characterized by autosomal dominant inheritance but de novo mutations have been reported. Clinical features of MODY include young-onset hyperglycemia, evidence of residual pancreatic function, and lack of beta cell autoimmunity or insulin resistance. Glucose-lowering medications are the main treatment options for MODY. The growing recognition of the clinical and public health significance of MODY by clinicians, researchers, and governments may lead to improved screening and diagnostic practices. Consequently, this review article aims to discuss the epidemiology, pathogenesis, diagnosis, and treatment of MODY based on relevant literature published from 1975 to 2020.

**Main body:**

The estimated prevalence of MODY from European cohorts is 1 per 10,000 in adults and 1 per 23,000 in children. Since little is known about the prevalence of MODY in African, Asian, South American, and Middle Eastern populations, further research in non-European cohorts is needed to help elucidate MODY’s exact prevalence. Currently, 14 distinct subtypes of MODY can be diagnosed through clinical assessment and genetic analysis. Various genetic mutations and disease mechanisms contribute to the pathogenesis of MODY. Management of MODY is subtype-specific and includes diet, oral antidiabetic drugs, or insulin.

**Conclusions:**

Incidence and prevalence estimates for MODY are derived from epidemiologic studies of young people with diabetes who live in Europe, Australia, and North America. Mechanisms involved in the pathogenesis of MODY include defective transcriptional regulation, abnormal metabolic enzymes, protein misfolding, dysfunctional ion channels, or impaired signal transduction. Clinicians should understand the epidemiology and pathogenesis of MODY because such knowledge is crucial for accurate diagnosis, individualized patient management, and screening of family members.

## Background

Maturity-onset diabetes of the young (MODY) is the most common type of monogenic diabetes, a clinically and genetically heterogeneous group of endocrine disorders resulting from mutations affecting a single gene involved in pancreatic beta cell function [1]. In 1975, Fajans and Tattersall used the acronym MODY for the first time in the literature to describe a cohort of patients with familial diabetes characterized by autosomal dominant inheritance of a primary defect in insulin secretion [2–4]. Currently, MODY accounts for 1–5% of all diabetes mellitus cases [5, 6]. Advancements in molecular diagnostics have led to the identification of 14 distinct subtypes of MODY thus far [7–11]. The most frequently occurring subtypes are attributed to mutations in genes encoding glucokinase (GCK) and hepatocyte nuclear factors (HNFs) [6, 12, 13]. Established treatment options for MODY include various glucose-lowering medications such as sulfonylureas, meglitinides, and insulin [9, 10]. MODY subtypes with progressive beta cell dysfunction may also require long-term management of diabetes-related complications.

The clinical and public health significance of MODY is increasingly being recognized by clinicians, researchers, and governments [14], which may help expand screening and genetic testing capacity. Consequently, this article aims to review current understanding of the epidemiology, pathogenesis, diagnosis, and treatment of MODY based on relevant literature published from 1975 to 2020.

### Diagnosis of MODY

**Fig. 1:**
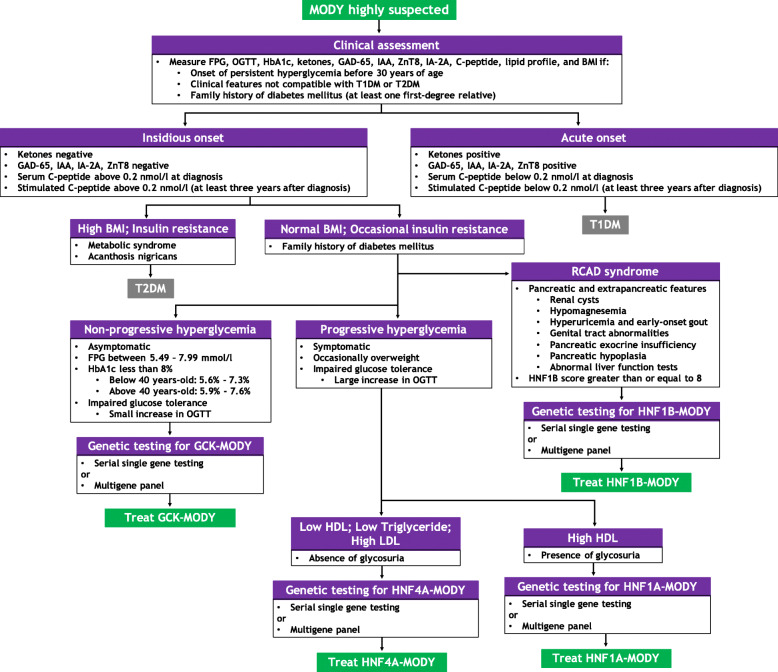
A systematic approach to the diagnosis of MODY. Diagnosis of MODY is a stepwise process guided by a high index of suspicion, clinical assessment, diabetes-specific tests, and comprehensive genetic testing. BMI: body mass index; FPG: fasting plasma glucose; GAD-65: glutamic acid decarboxylase-65 autoantibodies; HbA1c: glycated hemoglobin; HDL: high density lipoprotein; IAA: insulin autoantibodies; IA-2A: tyrosine phosphatase-related islet antigen-2 autoantibodies; LDL: low density lipoprotein; OADs: oral antidiabetic drugs; OGTT: oral glucose tolerance test; RCAD: renal cysts and diabetes; T1DM: type 1 diabetes mellitus; T2DM: type 2 diabetes mellitus; ZnT8: zinc transporter 8 autoantibodies

Early-onset diabetes, insulin independence, and autosomal dominant inheritance are traditionally associated with MODY [15]. Age of onset is particularly useful for distinguishing MODY from other types of diabetes. However, MODY subtypes with variable age of onset, low penetrance, or atypical presentation may not fulfill classical diagnostic criteria [15–18]. Furthermore, in a study involving 922 families referred for MODY testing, spontaneous de novo mutations affecting GCK, HNF1A, or HNF4A genes were reported in 11 of the 150 individuals who did not have autosomal dominant inheritance of diabetes mellitus or a multigenerational family history of hyperglycemia [19]. Refinement of the classical diagnostic triad may significantly increase clinical suspicion for MODY and facilitate the process of selecting and referring suspected patients for genetic testing [15, 17, 20]. The refined diagnostic criteria for MODY include: persistent hyperglycemia in early adulthood (typically before 30 years); clinical features incompatible with type 1 or type 2 diabetes mellitus (T1DM, T2DM); diabetes in at least one first-degree relative; evidence of residual pancreatic function; and absence of beta cell autoimmunity. Currently, there is no concise or standardized diagnostic algorithm for MODY [16]. To address this issue, a systematic approach to the diagnosis of common MODY subtypes is presented in Fig. 1. Overall, a diagnosis of MODY requires a high index of suspicion, clinical assessment, diabetes-specific tests, and comprehensive genetic testing [15, 21].

Clinical assessment and comprehensive genetic testing (serial single gene testing or multigene panel) are used to distinguish MODY from other types of diabetes mellitus, guide specific treatment, identify MODY mutations in family members with hyperglycemia, and reduce the risk of complications in asymptomatic family members [4, 21–23]. If genetic testing fails to identify one of the common MODY subtypes, comprehensive genomic testing (chromosomal microarray analysis or exome sequencing) can be used to diagnose rare pathogenic subtypes [24], characterize breakage points of whole gene deletions [25], and identify MODY in patients with clinical features suggestive of contiguous gene deletion syndrome [26, 27]. Nevertheless, the lack of a non-genetic biomarker that accurately identifies patients who have MODY, financial burdens related to cost of genetic tests, and limited access to specialized genetic testing facilities for patients and their families are challenges in clinical practice yet to be fully resolved.

### Epidemiology of MODY

#### Estimated incidence

MODY is usually diagnosed between the second and fifth decades of life [28], yet little is known about the global burden of the condition since population-based studies are seldom conducted. Table 1 presents a summary of the epidemiology of MODY based on available estimates from various countries. The minimum incidence of monogenic diabetes is estimated to be 0.2 cases per 100,000 children and youth below 18 years of age per year [41], while the estimated incidence of MODY is 2.4% in children and adolescents below 15 years of age with newly diagnosed diabetes mellitus [29]. There are a number of potential explanations for the scarcity of incidence estimates in the literature including: frequent misdiagnosis of MODY due to insidious onset of symptoms; overlap of clinical features with T1DM and T2DM; and low rates of referral for early comprehensive genetic testing due to lack of awareness of MODY among clinicians.

**Table 1:** Country-specific incidence and prevalence estimates of MODY

Country	Estimated incidence^**a**^	Estimated prevalence	References
**European region**			
Germany	2.4%	23.9 per million	[29, 30]
Netherlands	–	30 per million	[31, 32]
United Kingdom	–	68–108 per million	[31, 33]
Norway	–	0.94, 6.5%	[34, 35]
Sweden	–	1.2%	[36]
Lithuania	–	3.14%	[37]
Italy	–	5.5%	[38]
**Non-European region**			
United States	–	1.2%	[39]
Australia	–	89 per million	[40]

^a^The incidence cited is for children and adolescents below 15 years of age with newly diagnosed diabetes mellitus

#### Estimated prevalence

In contrast to incidence, the estimated prevalence of MODY is widely reported in the literature [31, 32, 39, 42]. The overall prevalence of MODY, mainly from European cohorts, is estimated to be 1–5 per 10,000 people, accounting for 1–5% of all diabetes mellitus cases [10, 28]. Current data suggests that prevalence of MODY varies by country [31]. Secondly, despite no reported ethnic predilection, prevalence of MODY varies by ethnicity [31], which may be due to disparities in availability and access to genetic testing [39]. Lastly, the estimated prevalence of MODY is 1 per 10,000 in adults and 1 per 23,000 in children [28], suggesting variation by age.

#### Prevalence of MODY in the European region

Due to well-maintained diabetes patient registries and centralized genetic testing facilities, MODY is well-studied in Europe, particularly the United Kingdom (UK), where monogenic diabetes has an estimated prevalence of 2.5% and the minimum prevalence of MODY is 68–108 cases per 1,000,000 [31, 33, 43, 44]. The most common subtypes in the UK are HNF1A-MODY (52% of all genetically confirmed cases), GCK-MODY (32%), HNF4A-MODY (10%), HNF1B-MODY (6%), and Neurogenic differentiation 1 (NEUROD1)-MODY or Insulin (INS)-MODY (less than 1%) [31, 33]. However, due to poor referral rates for genetic testing, approximately 80% of MODY cases in the UK are thought to be misdiagnosed as either T1DM or T2DM [33].

Prevalence estimates and MODY subtype distribution for young people living in Norway [34], Netherlands [31, 32], Germany [10, 30], and Poland [31, 45] are comparable to the UK. Importantly, the selection of participants for epidemiologic studies of MODY affects prevalence. For example, prevalence of genetically confirmed MODY is higher if autoantibody negative patients are recruited, such as in Norway (6.5%) [35], Sweden (1.2%) [36], and Lithuania (3.14%) [37], or if patients diagnosed with impaired fasting glycemia are included in studies, like in Italy (5.5%) [38].

#### Prevalence of MODY in non-European regions

While the prevalence of MODY in regions outside of Europe remains understudied, available reports suggest geographic and ethnic variation. In the United States, the estimated prevalence of MODY is 1.2% of all pediatric diabetes mellitus cases and the minimum prevalence of monogenic diabetes in youth below 20 years is estimated to be 21 per 1,000,000 [39]. In Western Australia on the other hand, the prevalence of MODY in diabetic patients below 35 years is 0.24%, which corresponds to an estimated minimum prevalence of 89 cases per 1,000,000 for the entire Australian population [40].

Unfortunately, the prevalence of MODY in African, Asian, South American, and Middle Eastern populations is not known. Clearly, research is needed to elucidate the exact prevalence of MODY in non-European regions. A recent study conducted in Brazil described the first case of NEUROD1-MODY in Latin America and identified a novel frameshift mutation [46], demonstrating that research in countries with multiethnic populations can enhance current understanding of the epidemiology and pathogenesis of MODY.

### Molecular pathogenesis and treatment of MODY

Various types of genetic mutations are implicated in the etiology of MODY [10, 47–49]. Table 2 presents the types of mutations associated with MODY and the affected chromosomes.

**Table 2:** MODY genes, chromosomal loci, and types of causative mutations

Gene	Chromosomal locus	Types of mutations	References
HNF4A	20q13.12	Missense, frameshift, splice site, nonsense, indel, deletion, insertion	[47, 49, 50]
HNF1A	12q24.31	Missense, frameshift, splice site, nonsense, indel, deletion, insertion	[47, 49, 51]
PDX1/IPF1	13q12.2	Missense, nonsense, deletion, insertion	[47, 49, 52]
HNF1B	17q12	Missense, frameshift, splice site, nonsense, indel, deletion, insertion	[47, 49, 53]
NEUROD1	2q32	Missense, frameshift, nonsense, indel, deletion, insertion	[47, 49, 54]
KLF11	2p25	Missense	[47, 49, 55]
PAX4	7q32	Missense, splice site, deletion	[47, 49, 56]
BLK	8p23	Missense	[47, 49, 57]
GCK	7p13	Missense, frameshift, splice site, nonsense, indel, deletion, insertion	[47, 49, 58]
CEL	9q34	Missense, frameshift, indel, deletion, insertion	[47, 49, 59]
INS	11p15.5	Missense, splice site, nonsense, indel, insertion	[47, 49, 60]
ABCC8	11p15	Missense	[47, 61, 62]
KCNJ11	11p15	Missense	[47, 63, 64]
APPL1	3p14.3	Missense, nonsense	[49, 65, 66]

Currently, MODY is classified based on the affected gene [67, 68]. However, a gene-based approach to classification assumes that MODY is one disease entity despite evidence of clinical, genetic, and pathophysiologic heterogeneity among the 14 recognized subtypes. In order to expand the current classification scheme, MODY subtypes can be organized into five categories according to their underlying molecular pathogenesis: transcriptional regulation disorders (dysfunctional nuclear transcription factors); enzyme disorders (dysfunctional or deficient metabolic enzymes); protein misfolding disorders; ion channel disorders (dysfunctional ion channels); and signal transduction disorders. A summary of the molecular pathogenesis and treatment options for MODY is presented in Table 3.

**Table 3:** Underlying molecular pathogenesis and treatment of MODY

MODY subtype	Pathophysiology	Treatment options	References
**Transcriptional regulation disorders**			
HNF4A (MODY1)	Beta cell dysfunction	Diet, sulfonylureas, insulin	[21, 69, 70]
HNF1A (MODY3)	Beta cell dysfunction	Diet, sulfonylureas, insulin, GLP-1 RAs	[69]
PDX1/IPF1 (MODY4)	Beta cell dysfunction	Diet, OADs, insulin	[21, 69]
HNF1B (MODY5)	Beta cell dysfunction	Insulin	[21, 69, 72]
NEUROD1 (MODY6)	Beta cell dysfunction	Diet, OADs, insulin	[21, 69]
KLF11 (MODY7)	Beta cell dysfunction	OADs, insulin	[21, 69]
PAX4 (MODY9)	Beta cell dysfunction	Diet, OADs, insulin	[21, 69]
BLK (MODY 11)	Insulin secretion defect	Diet, OADs, insulin	[21, 69]
**Enzyme disorders**			
GCK (MODY2)	Glucose sensing defect	Usually not treated. Insulin may be used during pregnancy	[69, 73, 74]
**Protein misfolding disorders**			
CEL (MODY8)	Pancreatic exocrine and endocrine dysfunction	OADs, insulin	[21, 69]
INS (MODY10)	Insulin biosynthesis defect	Diet, OADs, insulin	[21, 69]
**Ion channel disorders**			
ABCC8 (MODY12)	Insulin secretion defect	Sulfonylureas	[21, 69]
KCNJ11 (MODY13)	Insulin secretion defect	Sulfonylureas	[21, 69]
**Signal transduction disorders**			
APPL1 (MODY14)	Insulin secretion defect	Diet, OADs, insulin	[21, 69]

*OADs* oral antidiabetic drugs, *GLP-1 RAs* glucagon-like peptide-1 receptor agonists

### Transcriptional regulation disorders

#### HNF4A-MODY (subtype 1)

The hepatocyte nuclear factor 4 alpha (HNF4A) gene is expressed in liver, pancreatic islets cells and kidney. The HNF4A protein belongs to the steroid/thyroid hormone receptor superfamily of transcription factors and regulates expression of the HNF1A gene, hepatic gluconeogenesis, and lipoprotein biosynthesis [50]. Heterozygous mutations of this gene cause beta cell dysfunction, impaired glucose-stimulated insulin secretion, elevated low-density lipoprotein (LDL), and low levels of high-density lipoprotein (HDL) and triglyceride [47, 49, 50]. HNF4A-MODY is characterized by fetal macrosomia, transient neonatal hyperinsulinemic hypoglycemia, progressive development of hyperglycemia, and onset of diabetes mellitus in late adolescence or by 25 years of age. Due to the progressive nature of HNF4A-MODY, patients are susceptible to diabetes-related complications. Low carbohydrate diet or low-dose sulfonylureas are used to manage HNF4A-MODY initially but insulin therapy is usually required in advanced disease or during pregnancy [21, 69, 70]. The beneficial role of glucagon-like peptide-1 receptor agonists (GLP-1 RAs) in the management of patients with HNF4A-MODY has recently been reported [75]. Even though this finding is clinically significant, prospective studies are needed to help establish which patients should be offered incretin-based therapy.

#### HNF1A-MODY (subtype 3)

The hepatocyte nuclear factor 1 alpha (HNF1A) gene is expressed in liver, pancreas, kidney, and intestine [51, 68]. The HNF1A protein is a member of the homeodomain-containing superfamily of nuclear transcription factors and regulates the expression of the genes that encode insulin (INS), glucose transporter (GLUT) 1 and 2, and sodium/glucose cotransporter 2 (SGLT2) [51, 76]. Heterozygous mutations in the HNF1A gene cause progressive beta cell dysfunction, reduced glucose-stimulated insulin secretion, and low renal threshold for glucose (glycosuria) [47, 49, 51, 77]. HNF1A-MODY is typified by transient neonatal hyperinsulinemic hypoglycemia, progressive development of hyperglycemia during childhood, and onset of diabetes mellitus by 25 years of age. Patients with HNF1A-MODY are usually at risk of developing diabetes-related complications because glycemic control worsens over time. Low carbohydrate diet or low-dose sulfonylureas are used to manage HNF1A-MODY initially but insulin therapy is required in later stages of the disease or during pregnancy [21, 69, 70]. The use of GLP-1 RAs to effectively manage HNF1A-MODY has been reported [78, 79] and validated in a single-center randomized clinical trial [71, 80]. Therefore, clinicians may consider treating patients who have HNF1A-MODY with GLP-1 RAs, especially if episodes of hypoglycemia limit the tolerability of sulfonylureas.

#### PDX1/IPF1-MODY (subtype 4)

The pancreas/duodenum homeobox 1 (PDX1) protein, also known as IPF1 (insulin promoter factor 1), is a nuclear transcription factor that regulates pancreatic development and beta cell function by activating the expression of glucagon, INS, GLUT2, and GCK genes [49, 81]. Heterozygous mutations in the PDX1 gene lead to beta cell dysfunction and defective insulin secretion [47, 49, 52]. PDX1-MODY is characterized by diabetes mellitus with variable age of onset (usually between 17 and 67 years) compared to other MODY subtypes [1]. Management options for PDX1-MODY include diet, oral antidiabetic drugs (OADs), and insulin [21, 69].

#### HNF1B-MODY (subtype 5)

The hepatocyte nuclear factor 1 beta (HNF1B) protein, also known as transcription factor 2 (TCF2), is a member of the homeodomain-containing superfamily of nuclear transcription factors that regulates organogenesis of the pancreas, liver, genitourinary tract, kidney, intestine, and lungs [47, 82]. Heterozygous mutations cause beta cell dysfunction, hepatic insulin resistance, and a spectrum of congenital malformations termed renal cysts and diabetes (RCAD) syndrome [47, 49, 53, 83]. HNF1B-MODY exhibits variable multisystemic phenotypes characterized by a broad spectrum of pancreatic and extrapancreatic clinical manifestations [53, 84, 85]. Typically, renal disease occurs in childhood and diabetes mellitus arises in adolescence or early adulthood, with most affected individuals progressively requiring insulin. Management of HNF1B-MODY involves treatment of renal disease and early intensive insulin therapy to control hyperglycemia and delay the onset of microvascular complications [21, 69, 72].

#### NEUROD1-MODY (subtype 6)

The NEUROD1 gene is expressed in pancreatic and neuronal cells and the NEUROD1 protein is a basic helix-loop-helix nuclear transcription factor that regulates expression of the INS gene as well as pancreatic islet morphogenesis and neuronal development [54]. Heterozygous mutations in the NEUROD1 gene cause beta cell dysfunction [47, 49, 54]. NEUROD1-MODY is characterized by variable-onset diabetes mellitus with obese or non-obese phenotypes and occasional ketosis. Management options for NEUROD1-MODY include diet, OADs, or insulin [21, 69].

#### KLF11-MODY (subtype 7)

The krueppel-like factor 11 (KLF11) gene is expressed in pancreatic islets where it controls the expression of free radical scavengers such as catalase 1 and superoxide dismutase 2 [86]. The KLF11 protein is a zinc-finger nuclear transcription factor that controls beta cell function by acting as a glucose-inducible regulator of INS and PDX1 gene expression [55, 86, 87]. Heterozygous mutations in the KLF11 gene ultimately cause beta cell dysfunction and impaired insulin secretion [47, 49, 55, 86, 87]. Clinically, KLF11-MODY presents as early-onset diabetes mellitus and is managed with OADs or insulin [21, 69].

#### PAX4-MODY (subtype 9)

The paired box gene 4 (PAX4) protein is a nuclear transcription factor that regulates beta cell differentiation and represses the activity of INS and glucagon promoters [88]. Heterozygous mutations in the PAX4 gene cause abnormal beta cell development and lead to beta cell dysfunction and impaired glucose-stimulated insulin secretion [47, 49, 56]. PAX4-MODY is characterized by ketosis-prone diabetes mellitus [89] which is managed with diet, OADs, or insulin [21, 69].

#### BLK-MODY (subtype 11)

The B-lymphocyte specific tyrosine kinase (BLK) gene is a member of the SRC family of proto-oncogenes that is preferentially expressed in B-lymphocyte and beta cells [49, 57]. The BLK protein promotes insulin biosynthesis and secretion by upregulating the transcription factors PDX1 and NKX6.1 [57]. Heterozygous mutations in this gene attenuate BLK expression and/or activity resulting in PDX1 and NKX6.1 deficiency, defective glucose-stimulated insulin secretion, and reduced beta cell mass [47, 49, 57]. BLK-MODY manifests as diabetes mellitus with an overweight phenotype and is managed with diet, OADs, or insulin [21, 69].

### Enzyme disorders

#### GCK-MODY (subtype 2)

The GCK gene is expressed in liver and beta cells [49]. Glucokinase catalyzes adenosine triphosphate (ATP)-dependent phosphorylation of glucose to produce glucose-6-phosphate, which is the rate-limiting reaction of glucose metabolism. Heterozygous mutations may either reduce enzymatic activity [90–92] or promote glucokinase misfolding, aggregation, and degradation [93–95], leading to defective glucose sensing in beta cells, elevated threshold for glucose-stimulated insulin secretion, and impaired postprandial hepatic glycogen storage [47, 49, 58, 96]. GCK-MODY is generally non-progressive and characterized by preserved insulin secretion, mild fasting hyperglycemia present from birth, and minor postprandial glucose excursions. Consequently, this subtype is detected incidentally and treatment with glucose-lowering medications is unnecessary since patients tend to be asymptomatic [21, 69, 73, 97]. However, insulin is indicated during pregnancy to treat maternal hyperglycemia and reduce the risk of fetal macrosomia if serial ultrasound biometry suggests that the fetus has not inherited the maternal GCK mutation [69, 70, 74, 97, 98].

### Protein misfolding disorders

#### CEL-MODY (subtype 8)

The carboxyl-ester lipase (CEL) gene is expressed in acinar cells of the exocrine pancreas where it encodes a bile salt-dependent lipase that is incorporated into pancreatic digestive juice. The carboxyl-ester lipase hydrolyzes cholesterol esters and lipid soluble vitamins in the small intestine. Heterozygous mutations in the CEL gene are associated with early pancreatic atrophy and subsequent exocrine insufficiency, pancreatic lipomatosis, and endocrine dysfunction due to misfolding and cytotoxic aggregation of carboxyl-ester lipase [47, 49, 59, 99–101]. CEL-MODY presents as adulthood-onset diabetes mellitus and is treated with OADs or insulin [21, 69].

#### INS-MODY (subtype 10)

INS-MODY is caused by heterozygous mutations in the gene that encodes preproinsulin, the biologically inactive precursor of the insulin protein. Mutations in the INS gene cause severe misfolding and intracellular accumulation of proinsulin, defective nuclear factor-kappa-light-chain-enhancer of activated B cells (NF-kappa B), and abnormal insulin biosynthesis [47, 49, 60]. Consequently, prolonged endoplasmic reticulum stress activates the terminal unfolded protein response and leads to the induction of beta cell apoptosis. INS-MODY is characterized by reduced beta cell mass, progressive loss of insulin secretion, and variable-onset diabetes mellitus that is managed with diet, OADs, or insulin [21, 69].

### Ion channel disorders

#### ABCC8-MODY (subtype 12)

The ATP-binding cassette transporter subfamily C member 8 (ABCC8) gene is expressed in the pancreas where it controls the expression of the sulfonylurea receptor 1 (SUR1) subunit of the ATP-sensitive potassium channel found on the beta cell membrane. Opening and closing of the potassium channel regulates glucose-stimulated insulin secretion by coupling blood glucose levels and intracellular ATP concentration to the beta cell membrane’s electrical activity. Heterozygous mutations affecting the ABCC8 gene disrupt the potassium channel’s normal function leading to impaired insulin secretion [47, 61, 62, 102]. ABCC8-MODY may be characterized by congenital hypoglycemic hyperinsulinism, a transient or permanent form of neonatal diabetes mellitus, or adulthood-onset diabetes mellitus [61, 62, 102]. Sulfonylureas are the standard treatment option for ABCC8-MODY [21, 69].

#### KCNJ11-MODY (subtype 13)

The potassium channel inwardly rectifying subfamily J member 11 (KCNJ11) gene is highly expressed in the pancreas where it encodes the pore-forming Kir6.2 subunit of the ATP-sensitive potassium channel. Similar to ABCC8-MODY, heterozygous mutations in the KCNJ11 cause defective glucose-stimulated insulin secretion by disrupting the activity of the potassium channel [47, 63, 64, 102]. KCNJ11-MODY may be characterized by congenital hypoglycemic hyperinsulinism, a transient or permanent form of neonatal diabetes mellitus, or late-onset diabetes mellitus [63, 64, 102]. Sulfonylureas are the treatment of choice for KCNJ11-MODY [21, 69].

### Signal transduction disorders

#### APPL1-MODY (subtype 14)

The adaptor protein, phosphotyrosine interaction, PH domain, and leucine zipper-containing protein 1 (APPL1) gene is highly expressed in skeletal muscle, pancreas, liver, and adipose tissue. The APPL1 protein propagates the insulin signal within cells by interacting with key mediators including Akt serine/threonine kinase 2 (Akt2), insulin receptor substrate (IRS)-1 and IRS-2, and the insulin receptor [65, 103–106]. Additionally, APPL1 controls insulin-stimulated glucose uptake in skeletal muscle and adipose tissue [65, 105]. Heterozygous loss-of-function mutations in this gene lead to defective glucose-stimulated insulin secretion and reduced survival of beta cells [49, 65, 66, 106]. APPL1-MODY is a rare subtype and management options include diet, OADs, and insulin [21, 69].

## Conclusions

In conclusion, MODY accounts for 1–5% of all diabetes mellitus cases. Clinical assessment and comprehensive genetic testing are used to diagnose and classify MODY. Incidence and prevalence estimates for MODY are derived from epidemiologic studies of young people with diabetes who live in Europe, Australia, and North America. Mechanisms involved in the pathogenesis of MODY include defective transcriptional regulation, abnormal metabolic enzymes, protein misfolding, dysfunctional ion channels, or impaired signal transduction. Management of MODY is subtype-specific and established treatment options include diet, oral antidiabetic drugs, or insulin. Clinicians should possess a thorough understanding of the epidemiology and pathogenesis of MODY in order to accurately diagnose patients, individualize patient management and follow-up, and screen family members of affected individuals for diabetes mellitus.

## Data Availability

Not applicable.

## Abbreviations

ABCC8: ATP-binding cassette transporter subfamily C member 8
Akt2: Akt serine/threonine kinase 2
APPL1: Adaptor protein, phosphotyrosine interaction, PH domain, and leucine zipper-containing protein 1
ATP: Adenosine triphosphate
BLK: B-lymphocyte specific tyrosine kinase
CEL: Carboxyl-ester lipase
GCK: Glucokinase
GLP-1 RAs: Glucagon-like peptide-1 receptor agonists
GLUT: Glucose transporter
HNFs: Hepatocyte nuclear factors
HNF1A: Hepatocyte nuclear factor 1 alpha
HNF4A: Hepatocyte Nuclear Factor 4 alpha
HNF1B: Hepatocyte Nuclear Factor 1 beta
HDL: High-density lipoprotein
INS: Insulin
IPF1: Insulin promoter factor 1
IRS: Insulin receptor substrate
KCNJ11: Potassium channel inwardly rectifying subfamily J member 11
KLF11: Krueppel-like factor 11
LDL: Low-density lipoprotein
NEUROD1: Neurogenic differentiation 1
NF-kappa B: Nuclear factor-kappa-light-chain-enhancer of activated B cells
MODY: Maturity-onset diabetes of the young
OADS: Oral antidiabetic drugs
PAX4: Paired box gene 4
PDX1: Pancreas/duodenum homeobox 1
RCAD: Renal cysts and diabetes
SGLT2: Sodium/glucose cotransporter 2
SUR 1: Sulfonylurea receptor 1
TCF2: Transcription factor 2
T1DM: Type 1 diabetes mellitus
T2DM: Type 2 diabetes mellitus
UK: United Kingdom
